# A Rare Case of Asymptomatic Insulinoma with Mesocolonic Lymph Node Metastases and Long-Term Stability

**DOI:** 10.70352/scrj.cr.25-0518

**Published:** 2026-02-27

**Authors:** Chie Kitami, Yasuyuki Kawachi, Atsushi Nishimura, Tetsuya Nakano, Shigeto Makino, Mikako Kawahara

**Affiliations:** Department of Surgery, Nagaoka Chuo General Hospital, Nagaoka, Niigata, Japan

**Keywords:** insulinoma, pancreatic neuroendocrine tumor, lymph node metastasis, selective arterial calcium injection, intraoperative insulin monitoring

## Abstract

**INTRODUCTION:**

Malignant insulinomas are rare, and lymph node metastases are particularly uncommon in small, low-grade tumors. We report an asymptomatic insulinoma of the pancreatic body with multiple lymph node metastases confined to the transverse mesocolon, which remained radiologically stable for at least 3 years prior to the diagnosis. This case highlights the potential for metastatic disease, even in indolent insulinomas.

**CASE PRESENTATION:**

A 75-year-old man was referred after repeated findings of low fasting glucose levels during annual health screenings over 3 years. Despite persistent hypoglycemia, the patient remained asymptomatic and untreated. On admission, the fasting blood glucose level was 45 mg/dL, the immunoreactive insulin level was 8.7 μU/mL, and the serum C-peptide level was 2.0 ng/mL, with insulin secretion indices within normal limits. Dynamic contrast-enhanced CT revealed a 1-cm hypervascular lesion in the pancreatic body and 3 similar lesions in the transverse mesocolon. A retrospective review of earlier scans confirmed their long-term stability. Selective arterial calcium injection testing revealed insulin secretion from both the dorsal pancreatic and accessory middle colic arteries, corresponding to the pancreatic and mesocolonic lesions, respectively. Central pancreatectomy with en bloc mesocolon resection was performed. Intraoperative portal venous insulin levels declined from 102 μU/mL before resection to 10 μU/mL before closure, confirming the complete tumor removal. Histopathological analysis revealed a well-differentiated neuroendocrine tumor composed of islet cell–like neoplastic cells with a Ki-67 labeling index below 1%. Four metastatic lymph nodes were identified in the patient. The patient has remained recurrence-free for 7 years, with normal fasting glucose and insulin levels.

**CONCLUSIONS:**

This case demonstrates that even small, low-grade insulinomas can metastasize to the lymph nodes through atypical drainage pathways. Favorable tumor biology may mitigate the adverse prognostic implications of nodal disease in well-differentiated pancreatic neuroendocrine tumors. Comprehensive lymph node assessment combined with functional localization techniques, such as selective arterial calcium injection testing and intraoperative insulin monitoring, may be essential for achieving curative resection and long-term disease control.

## Abbreviations

DPA
: dorsal pancreatic artery
FBG
: fasting blood glucose
IRI
: immunoreactive insulin
isCGM
: intermittently scanned continuous glucose nonitoring
pNET
: pancreatic neuroendocrine tumors
SACI
: selective arterial calcium injection
SMA
: superior mesenteric artery

## INTRODUCTION

Insulinoma is the most common functional pNET. It is typically small, solitary, and benign, with an incidence of 1–4 cases per million people per year.^1)^ Most insulinomas are benign, solitary, and <2 cm in diameter; however, approximately 5%–10% are malignant, defined by the presence of metastases or local invasion.^2)^ Among malignant cases, the liver is the most common site of metastasis, accounting for approximately 80% of distant spread, whereas lymph node involvement is relatively uncommon.^3,4)^

Preoperative identification of lymph node metastasis remains challenging because of the small size of metastatic nodes and the often subtle clinical course of the disease. When the primary tumor is <2 cm, nodal metastasis is considered rare, and enucleation is frequently deemed sufficient.^5,6)^ Nevertheless, advanced imaging modalities, such as contrast-enhanced CT and SACI testing, can enhance diagnostic accuracy and inform surgical strategy. Here, we report an exceptionally rare case of an asymptomatic insulinoma of the pancreatic body with multiple lymph node metastases confined to the transverse mesocolon, which was radiologically detectable but stable for at least 3 years prior to diagnosis. This case is also notable for the atypical lymphatic drainage route, preoperative localization using SACI, and intraoperative confirmation of complete resection via portal venous IRI monitoring. We discuss the potential lymphatic pathways, prognostic implications of nodal spread in low-grade pNETs, and the role of multimodal functional assessment in surgical management.

## CASE PRESENTATION

A 75-year-old man was referred to our hospital for the evaluation of asymptomatic hypoglycemia. Annual health checkups over the previous 3 years had repeatedly shown low fasting plasma glucose levels. Despite these abnormalities, the patient experienced no subjective symptoms and did not seek medical attention. Following strong recommendations, the patient presented for further evaluation. He was an avid mountain climber and reported no episodes suggestive of hypoglycemia, even during strenuous physical activities. The patient had presented 3 years earlier for evaluation of chronic cough. At that time, the FBG level was 58 mg/dL; however, only an evaluation for chronic cough was performed, and hypoglycemia was not further investigated. The CT scan was considered unremarkable at that time.

At his initial visit, the FBG level was 45 mg/dL, the IRI level was 8.7 μU/mL, and the serum C-peptide level was 2.0 ng/mL. Indices of autonomous insulin secretion were within normal limits: Fajans’ index (IRI/FBG) 0.19 (reference ≤0.3), Grunt’s index (FBG/IRI) 5.1 (reference ≥2.5), and Turner’s index (IRI × 100/[FBG − 30]) 58 (reference ≤200).

**Fig. 1:**
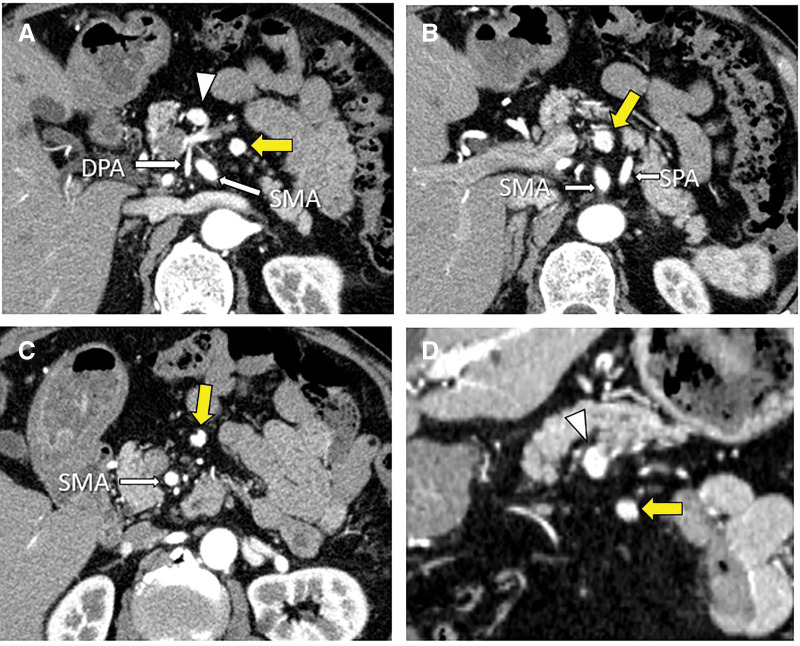
Dynamic contrast-enhanced CT demonstrating a 10-mm hyperenhancing lesion in the pancreatic body (white arrowhead) (**A**), and 3 similarly enhancing nodules in the transverse mesocolon (yellow arrows) (**B** and **C**). (**D**) Coronal section showing the tumor (arrowhead) and a lymph node (arrow). DPA, dorsal pancreatic artery; SMA, superior mesenteric artery; SPA, splenic artery

**Fig. 2:**
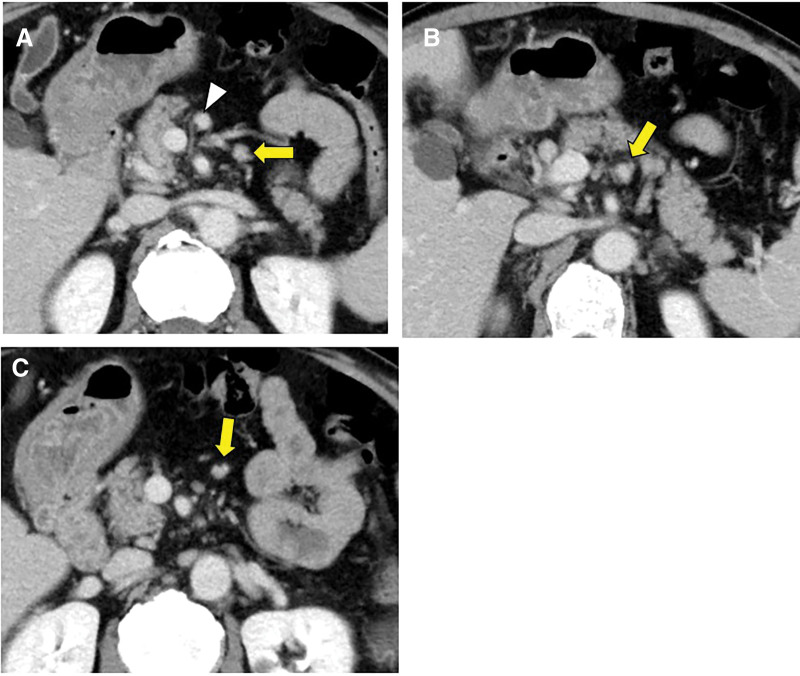
Contrast-enhanced CT obtained 3 years prior to diagnosis for evaluation of chronic cough revealed a 10-mm enhancing lesion in the pancreatic body (white arrowhead) (**A**) and 3 nodules in the transverse mesocolon (yellow arrows) (**B** and **C**).

**Fig. 3:**
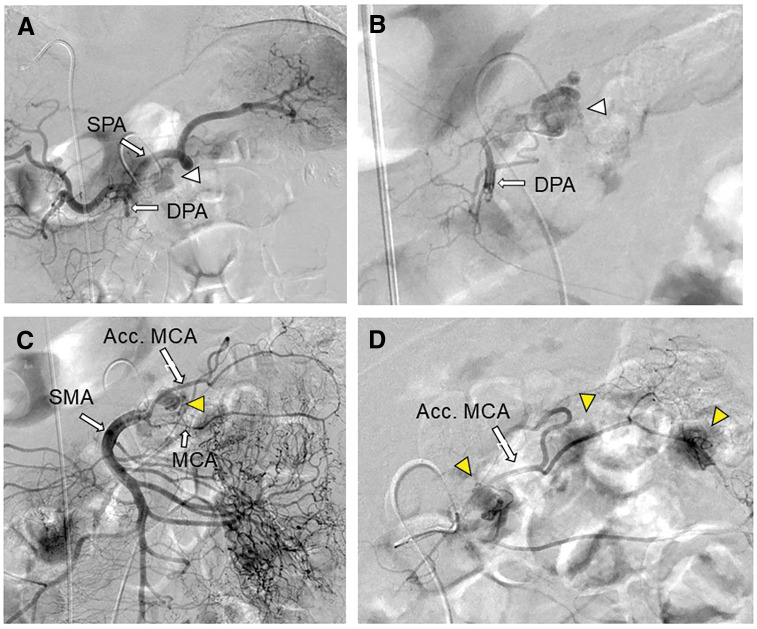
Angiographic findings during the selective arterial calcium injection test. On angiography, the primary lesion was visualized via the dorsal pancreatic artery (white arrowhead) (**A**, **B**), and 3 hypervascular lesions were detected via the accessory middle colic artery (yellow arrowheads) (**C**, **D**). Acc. MCA, accessory middle colic artery; DPA, dorsal pancreatic artery; MCA, middle colic artery; SMA, superior mesenteric artery; SPA, splenic artery

**Fig. 4:**
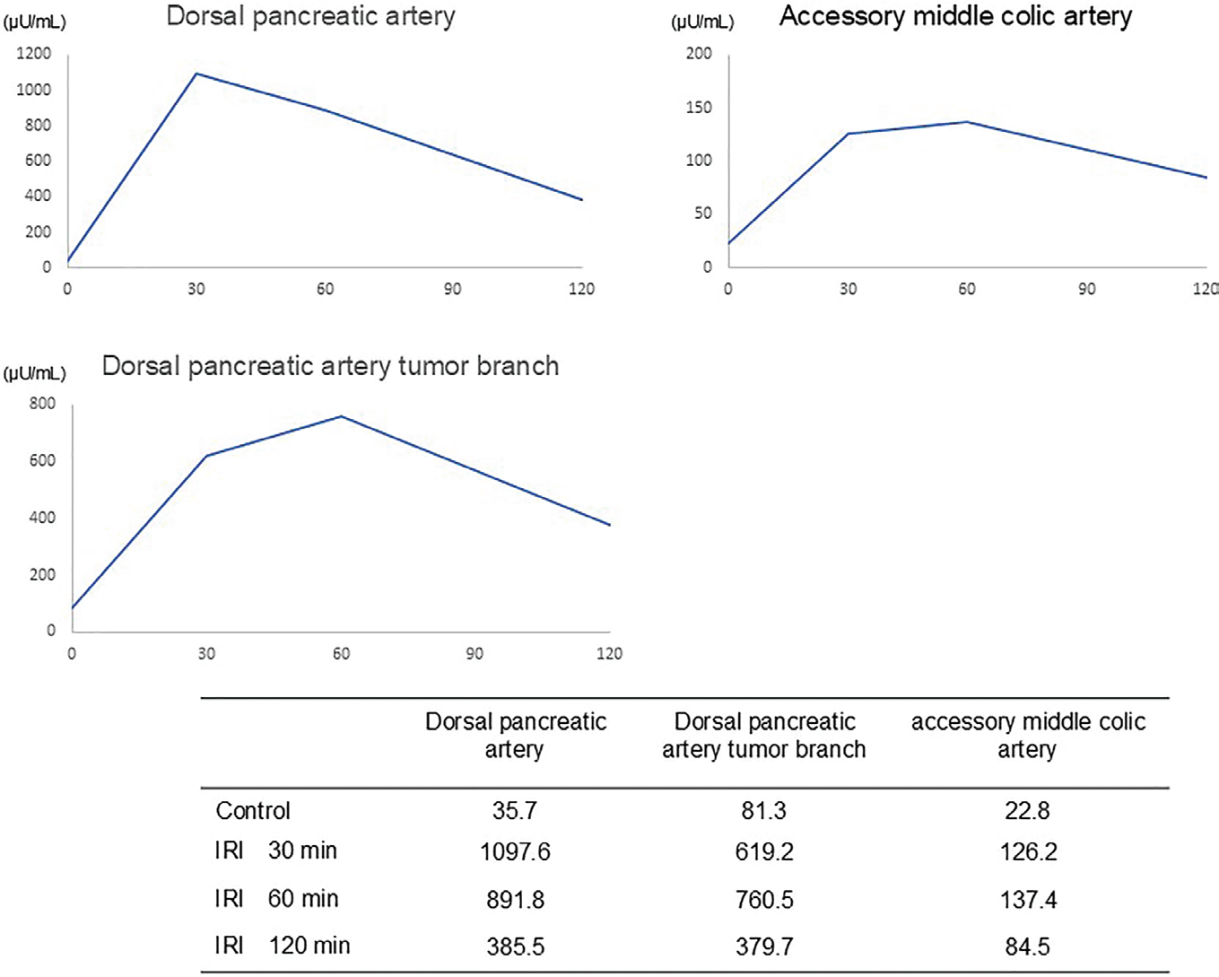
Changes in hepatic venous IRI (μU/mL) during the selective arterial calcium injection test. Marked step-up of IRI levels after injection into the dorsal pancreatic artery and the accessory middle colic artery. IRI, immunoreactive insulin

**Fig. 5:**
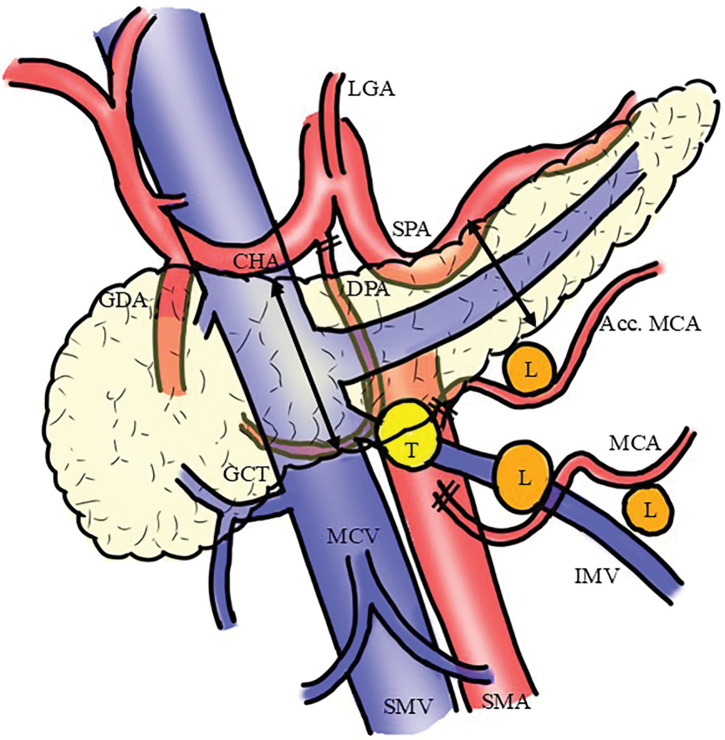
Surgical schema. Acc. MCA, accessory middle colic artery; CHA, common hepatic artery; DPA, dorsal pancreatic artery; GCT, gastrocolic trunk; GDA, gastroduodenal artery; IMV, inferior mesenteric vein; L, metastatic lymph node; LGA, left gastric artery; MCA, middle colic artery; MCV, middle colic vein; SMA, superior mesenteric artery; SMV, superior mesenteric vein; SPA, splenic artery; T, primary tumor

Dynamic contrast-enhanced CT revealed a 10-mm hyperenhancing lesion protruding from the pancreatic body, along with 3 similarly enhancing nodules within the transverse mesocolon (**Fig. 1**). A retrospective review 3 years prior demonstrated a 10-mm hypervascular lesion in the pancreatic body and 3 nodules within the transverse mesocolon, all unchanged in size over 3 years (**Fig. 2**). During the SACI test, angiography revealed the primary tumor supplied by the DPA, along with 3 additional hypervascular foci supplied by the accessory middle colic artery (**Fig. 3**). The SACI test demonstrated positive step-up responses in insulin levels from the DPA (corresponding to the pancreatic lesion) and the accessory middle colic artery (corresponding to the mesocolonic nodules), supporting the diagnosis of multiple insulin-secreting lesions (**Fig. 4**). Based on these findings, a laparotomy was performed under the preoperative diagnosis of insulinoma of the pancreatic body with lymph node metastases in the mesocolon. No gross mass was evident on inspection; however, palpation revealed nodules along the inferior border of the pancreatic body and within the transverse mesocolon. Central pancreatectomy was performed with the intent of systematic resection along the route from the pancreas to the mesocolon. The transverse mesocolon was resected en bloc, while preserving the marginal vessels. The accessory middle colic artery, which arose from the SMA, was ligated and divided. Proximally, the pancreas was transected just above the portal vein and carefully dissected from the portal and superior mesenteric veins, preserving the splenic artery and vein (**Fig. 5**). Reconstruction was achieved using Roux-en-Y pancreaticojejunostomy with duct-to-mucosa anastomosis at the distal pancreatic remnant. For intraoperative monitoring, a catheter was inserted into the middle colic vein near the junction of the portal and splenic veins. Portal venous IRI levels decreased from 102 μU/mL pre-resection to 24 μU/mL immediately after resection, and to 10 μU/mL prior to abdominal closure (**Table 1**). The operative time was 4 hours and 30 minutes, with an estimated blood loss of 130 mL. Postoperative complications included a pancreatic fistula classified as International Study Group of Pancreatic Fistula Grade B, and the patient was discharged on POD 21.

**Table 1:** Surgical case reports of insulinoma with lymph node metastasis

Author	Year	Age	Sex	Synchronous/metachronous	Primary tumor size (mm)	Ki-67 labeling index	Outcome	
Walker	2022	36	F	Met	16	3.8	8 Y alive	
Murase	2004	57	M	Syn	23	N.A.	10 M alive	
Hirshberg	2005	47	F	Syn	40	N.A.	24 Y alive	
	2005	68	F	Syn	80	N.A.	25 Y alive	
	2005	30	M	Syn	90	N.A.	23 Y alive	
	2005	33	F	Syn	15	N.A.	10 Y alive	
Ueda	2016	71	M	Syn	15	20	2 Y 3 M alive	
Lee	2003	53	F	Syn	58	13	18 M alive	
Tarris	2023	38	F	Syn	90	<2	6 M alive	
Sada	2020	Mean 56	N.A.	Syn	Mean 40	N.A.	5-year CSS 81.5%	Node positive: 15/77 (34%)*

*Among 77 cases, 15 (34%) were node positive.

CSS, cancer-specific survival; F, female; M, male; M, months; Met, metachronous; N.A., not available; Syn, synchronous; Y, years

**Fig. 6:**
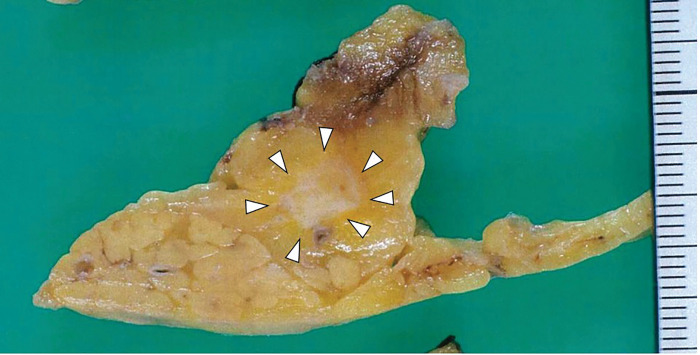
Gross pathology showing a solid, pale, well-circumscribed tumor in the pancreatic body, measuring 10 × 8 mm (white arrowheads).

**Fig. 7:**
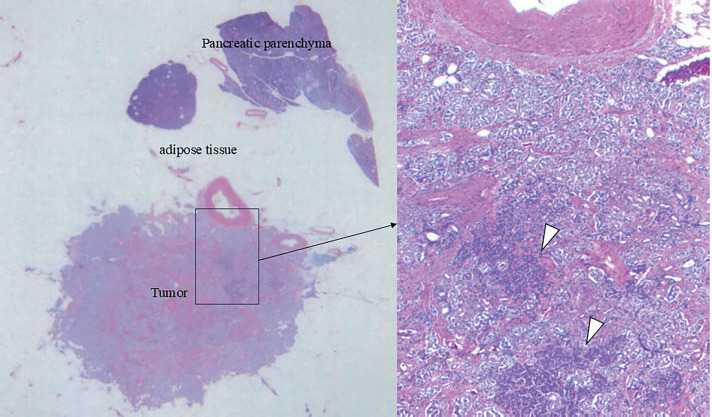
Adipose tissue was interposed between the pancreatic parenchyma and the tumor presumed to represent the primary lesion, and direct continuity could not be demonstrated. Foci of normal pancreatic tissue (white arrowheads) were observed within portions of the tumor.

**Fig. 8:**
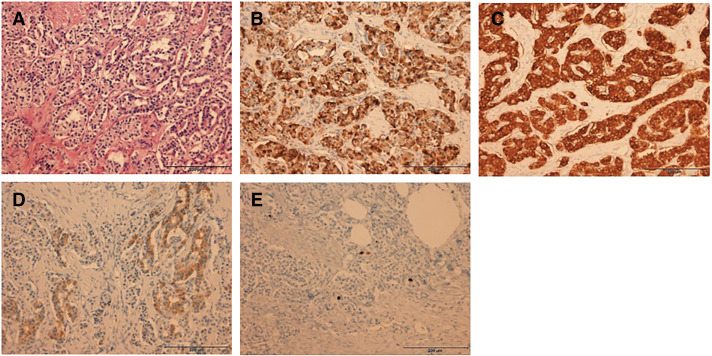
Histopathological findings of the pancreatic lesion. Hematoxylin–eosin staining revealed nests of uniform cells with round nuclei and pale cytoplasm. Immunohistochemical examinations were positive for chromogranin A, synaptophysin, and insulin, with a Ki-67 labeling index of <1%. (**A**) Hematoxylin–eosin staining, (**B**) chromogranin A, (**C**) synaptophysin, (**D**) insulin, and (**E**) Ki-67.

**Fig. 9:**
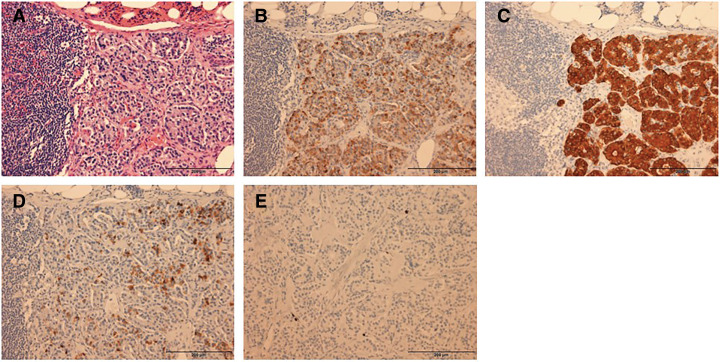
Histopathological findings of the lymph nodes. (**A**) Hematoxylin–eosin staining, (**B**) chromogranin (**A**, **C**) synaptophysin, (**D**) insulin, and (**E**) Ki-67. Demonstrate similar pathological characteristics to the primary lesion.

Gross pathological examination revealed a 10 × 8-mm solid, pale tumor in the pancreatic body (**Fig. 6**). Histologically, hematoxylin–eosin staining revealed nests of islet cell-like neoplastic cells with round nuclei and pale cytoplasm. Four metastatic lesions were identified in the mesocolonic lymph nodes. Continuity with the pancreatic parenchyma could not be confirmed in the resected specimen, and the possibility that the insulinoma arose from the mesocolon cannot be entirely excluded. However, because foci of normal pancreatic tissue were identified within the tumor and the lesion received its blood supply from the DPA, it was considered to be of pancreatic origin (**Fig. 7**). Immunohistochemical analysis revealed that both primary and metastatic lesions were positive for chromogranin A, synaptophysin, and insulin. The Ki-67 labeling index was <1%. The histopathological findings of the primary lesion and lymph nodes are shown in **Figs. 8** and **9**, respectively.

The patient remained disease-free for 7 years postoperatively, with normal fasting glucose and insulin levels and no evidence of recurrence.

## DISCUSSION

This case describes an incidentally detected, asymptomatic insulinoma with preoperative imaging evidence of lymph node metastasis in the transverse mesocolon. Although more than 90% of insulinomas are benign, malignant forms with metastatic potential occur in approximately 5%–10% of cases.^2,3)^ The liver is the most frequent site of distant spread, whereas lymph node involvement is relatively uncommon. In a series of 791 cases, Soga et al.^3)^ reported an overall metastasis rate of 7.2%, with hepatic and nodal metastases in 70.2% and 50.9% of metastatic cases, respectively. It is particularly unusual for tumors smaller than 1 cm, especially those with a low proliferative index (Ki-67 <1%), to exhibit nodal involvement. Tsutsumi et al.^7)^ and Tanaka et al.^8)^ have reported that such small, well-differentiated pNETs carry a very low risk of lymph node metastases.

A systematic PubMed search using the keywords “insulinoma,” “lymph node metastasis,” and “resection” identified cases with detailed clinical descriptions^9–15)^ (**Table 1**). The mean patient age was 48.9 years; except for a single case, all presentations were synchronous. The mean primary tumor size was 46.7 mm, larger than that observed in the present case, yet clinical outcomes across all reported cases were favorable. Although nodal metastasis is often regarded as a negative prognostic factor, several studies suggest that in well-differentiated, low-grade tumors, it may not significantly affect long-term survival. Walker et al.^9)^ demonstrated favorable outcomes even in a patient with isolated nodal recurrence. In our case, despite multiple lymph node metastases from a 10-mm tumor with Ki-67 <1%, no recurrence was observed over 7 years. This suggests that favorable tumor biology, particularly a very low proliferative index, may be more influential than nodal status in determining prognosis. These findings highlight the need for individualized oncologic assessment in pNETs.

The lymphatic drainage of the lower pancreas typically proceeds toward the lymph nodes surrounding the SMA and subsequently to the para-aortic region. However, the precise pathways of pancreatic body tumors remain incompletely characterized.^16)^ In the present case, lymph node metastases were located within the transverse mesocolon, suggesting an atypical drainage route. Anatomically, the pancreatic body lies in close proximity to the transverse mesocolon, and previous studies have demonstrated vascular and lymphatic connections between these regions. Stelzner et al.^17)^ described aberrant lymphatic pathways from the transverse colon to the pancreatic and gastroepiploic lymph nodes, facilitated by small vessels traversing the transverse mesocolon and terminating near the lower pancreatic border. These connections may permit retrograde or lateral lymphatic spread in pancreatic malignancy. Mizutani et al.^18)^ further reported that advanced pancreatic body and tail cancers frequently invade retroperitoneal tissues, including the root of the transverse mesocolon, and emphasized the oncologic value of en bloc resection of this region using a mesenteric approach. Additionally, indocyanine green fluorescence studies have demonstrated multiple lymphatic routes from the pancreas, including pathways along the middle colic artery toward the transverse colon and para-aortic nodes.^19)^ In this case, metastatic nodes were present within this atypical basin, simple enucleation was deemed oncologically insufficient, leading us to perform central pancreatectomy with en bloc resection of the transverse mesocolon.

Another notable aspect of this case was the absence of symptoms despite persistent fasting hypoglycemia for over 3 years. This phenomenon, known as hypoglycemia unawareness, occurs when the counterregulatory hormone response becomes downregulated after prolonged hypoglycemia, thereby attenuating sympathetic warning signs.^20)^ Mitrakou et al.^21)^ demonstrated that this condition is reversible in patients with insulinomas, but emphasized its clinical importance because it can significantly delay diagnosis despite long-standing biochemical hypoglycemia. This mechanism likely contributed to the delayed diagnosis in our patient.

Intraoperative monitoring of IRI levels is a sensitive method for confirming the complete resection of insulinomas, particularly in small or multifocal diseases.^22)^ In our case, portal venous IRI levels dropped markedly after tumor removal, consistent with previous studies demonstrating that rapid intraoperative IRI decline correlates with biochemical remission. Muneoka et al.^23)^ also emphasized that a rapid IRI decline correlates with biochemical remission, guiding surgical decisions when imaging is inconclusive. Although CT and SACI correctly identified 3 metastatic nodes preoperatively, additional histologically positive nodes were found only after systematic mesocolon resection in this case. Intraoperative IRI monitoring was valuable for confirming complete tumor removal. SACI remains a cornerstone of preoperative localization, particularly when conventional imaging fails to detect small or deep-seated lesions.^24)^ SACI remains a cornerstone for localizing small or deep-seated insulinomas, with detection rates exceeding those of CT, MRI, and endoscopic US.^25,26)^ Recently, isCGM has also been introduced as an intraoperative adjunct, providing real-time glucose trends and aiding surgical decision-making, especially in patients with hypoglycemia unawareness.^27,28)^ The integration of intraoperative IRI monitoring, SACI, and isCGM provides a robust multimodal approach for accurate localization and functional assessment in atypical presentations such as this case.

## CONCLUSIONS

We report a rare case of an asymptomatic, radiologically stable 10-mm insulinoma with multiple lymph node metastases confined to the transverse mesocolon. Despite prolonged biochemical hypoglycemia, clinical symptoms were absent due to hypoglycemia unawareness. The absence of recurrence over 7 years suggests that favorable tumor biology may mitigate the prognostic impact of nodal metastasis in well-differentiated pNETs. This case emphasizes the importance of recognizing atypical lymphatic spread, considering systematic assessment of lymphatic basins even in small insulinomas, and employing multimodal functional localization to achieve complete resection.

## DECLARATIONS

### Availability of data and material

The dataset supporting the conclusions of this article is included within the article.
